# Identification of Novel Mutation in the *ABCA12* Gene Causing Harlequin Ichthyosis

**DOI:** 10.1002/ccr3.72010

**Published:** 2026-02-06

**Authors:** Nadia Soltani, Zahra Bayati, Mohsen Soosanabadi, Akbar Zamani, Asghar Lotfi, Milad Gholami

**Affiliations:** ^1^ Student Research Committee, School of Medicine Arak University of Medical Sciences Arak Iran; ^2^ Department of Biology, Portion of Genetic Arak University Arak Iran; ^3^ Department of Medical Genetics Semnan University of Medical Sciences Semnan Iran; ^4^ Department of Pediatrics, Amir‐Kabir Hospital Arak University of Medical Sciences Arak Iran; ^5^ Department of Biochemistry and Genetics, School of Medicine Arak University of Medical Sciences Arak Iran

**Keywords:** *ABCA12*, harlequin ichthyosis, mutation, whole exome sequencing

## Abstract

Harlequin ichthyosis (HI) is an uncommon and extremely severe hereditary condition that primarily affects the skin. Infants born with this disorder display dense skin and prominent diamond‐shaped plates that cover a significant portion of their bodies. Infants with this disease have difficulty regulating body temperature and maintaining hydration, leading to respiratory failure and feeding problems, making them more vulnerable to infections. Most patients die shortly after birth because of these clinical symptoms. Scientific evidence has shown that a mutation in the *ABCA12* gene is the principal underlying cause of HI. Using whole‐exome sequencing, we identified a novel mutation in an Iranian infant with HI. This case presented with characteristic cutaneous manifestations, leading to the discovery of a novel homozygous mutation in the *ABCA12* gene. This specific mutation [c.4702_4706del, p.(Leu1568IlefsTer5)] has not been reported in any other cases of harlequin ichthyosis and was detected in a heterozygous state in asymptomatic parents. The insights gained from analyzing this family enhance our understanding of the disease's molecular origin, aid in carrier identification, support genetic counseling, and emphasize the importance of prenatal genetic screening for families with a history of HI.

## Key Clinical Message

Harlequin ichthyosis is a severe genetic skin disease caused by mutations in the *ABCA12* gene. This study identifies a novel homozygous mutation in an Iranian infant. The asymptomatic parents were heterozygous carriers. The findings enhance disease understanding, facilitate carrier detection, and highlight the need for genetic counseling and prenatal screening.

## 1 Introduction

Autosomal recessive congenital ichthyosis (ARCI) is a genetically and phenotypically heterogeneous group of disorders characterized by hyperkeratosis in addition to dry, scaly skin [1]. In patients with ARCI, three main phenotypes have been identified: harlequin ichthyosis, congenital ichthyosiform erythroderma, and lamellar ichthyosis [2]. Among the various types of ichthyosis, HI is a severe and often fatal subtype, with the highest mortality rate among affected newborns [3, 4]. The incidence of HI is approximately 1 in 300,000 live births, making it a rare disease with significant clinical implications [5, 6]. These patients typically exhibit significant clinical manifestations at birth, such as thick, large skin scales covering the entire body and separated by deep polygonal fissures along with distinct features, such as everted eyelids, lips, and flattened ears [3, 4, 7].

ARCI is attributed to biallelic mutations in a variety of genes, including *TGM1*, *ABCA12*, *CERS3*, *SDR9C7*, *SULT2B1*, *NIPAL4*, *PNPLA1*, *CASP14*, *ALOXE3*, *CYP4F22*, *LIPN*, and *ALOX12B* [1, 8, 9, 10, 11, 12, 13, 14, 15]. Among these, the main gene associated with ARCI is *ABCA12*, which can lead to different subtypes depending on the specific type of mutation. Truncating mutations in *ABCA12* are primarily linked to HI, whereas milder phenotypes, such as congenital ichthyosiform erythroderma (CIE) or lamellar ichthyosis (LI), are usually associated with missense mutations in this gene [7, 16].

The *ABCA12* gene is located on chromosome 2q35, is approximately 207 kb long, and consists of 55 exons. The *ABCA12* gene codes for a 2595‐amino‐acid protein belonging to the ATP‐binding cassette (ABC) transporter superfamily [11, 17, 18]. ABC transporter proteins transport various biomolecules across cell membranes. This protein consists of two transmembrane (TM) domains, each with six hydrophobic transmembrane helices, and two cytoplasmic ATP‐binding cassette domains with highly conserved motifs such as Walker A and Walker B, which are essential for its transport function [17, 19, 20].

In skin cells, ABCA12 facilitates the transport of lipids, including glucosylceramide (GlcCer), via lamellar granules (LGs). This process culminates in the release of lipids onto the apical surface of granular keratinocytes, ultimately leading to the formation of lipid lamellae in the stratum corneum. ABCA12 is predominantly located in the membranes of the Golgi network and stratum corneum granules in the upper epidermis, and is mainly expressed in the upper squamous and granular cells that store lipids essential for skin barrier formation [17, 19, 20]. Mutations in *ABCA12* lead to disturbances in the secretion of proteases and lipids into the interstitial space between the cells of the stratum corneum. As a result, lipid droplets and abnormal vacuoles accumulate in cells that are not fully keratinized, except for those of the tongue, leading to the formation of dense granules or multilayered structures that give rise to the white dermal layer [17]. Insufficient lipid transport within the interstitial space gives rise to hyperkeratinization and inflammation in the epidermis, leading to a significant disruption of the epidermal barrier's function. Subsequently, this disruption causes a notable increase in transepidermal water loss, a defining trait observed in individuals with harlequin ichthyosis [21, 22]. The aim of this study was to perform a mutational analysis of an Iranian pedigree affected by Harlequin ichthyosis (HI) and a history of consanguineous marriage.

## 2 Materials and Methods

### 2.1 Family Recruitment and Ethical Statement

**FIGURE 1:**
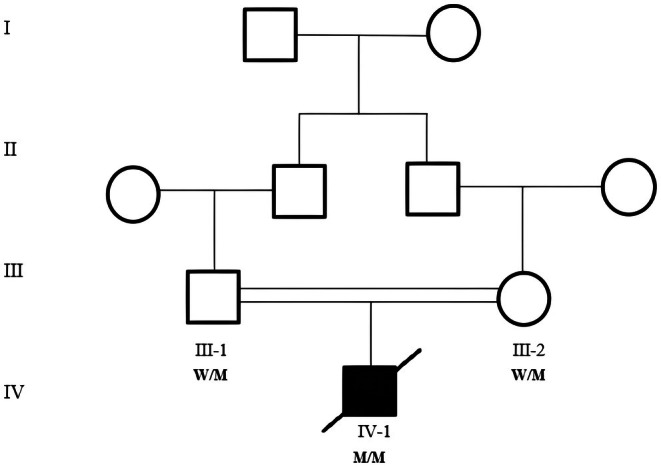
Family pedigree of Harlequin ichthyosis. Genotypes are indicated below: M/M (homozygous mutant), M/W (heterozygous carrier). The proband is homozygous (M/M) and deceased.

The proband (Figure 1) with Harlequin ichthyosis, who was born at the Amir‐Kabir Hospital in Arak, was included in this study. The parents provided written informed consent to participate in the study. This study was approved by the Ethics Committee of the Arak University of Medical Sciences, Arak, Iran (IR.ARAKMU.REC.1402.186).

### 2.2 Whole Exome Sequencing

Genomic DNA was extracted from 6 mL of peripheral blood using the salting‐out method. DNA quality was assessed using 1% agarose gel electrophoresis. Genomic DNA (gDNA) was used from the patient, and exome capture was performed using the twist‐v2.0 kit. Exons were sequenced to a target coverage of 100×. The NovaSeq 6000 platform was used to sequence enriched libraries. The sequencing data alignment, variant calling, annotation, variant prioritization, and pathogenicity prediction were performed.

### 2.3 Sanger Sequencing Validation

Primer sequences for exon 29 of *ABCA12* were designed using the Primer3Plus website (https://www.bioinformatics.nl/cgi‐bin/primer3plus/primer3plus.cgi), including the forward primer (5′‐ACAGCATTGGCCAGAAAAA‐3′) and reverse primer (5′‐GGCAGCTACCATTAAAATTAGCAA‐3′), yielding a 230 bp product. The total PCR reaction volume was 50 μL, containing 24 μL of PCR Master Mix, 1 μL of each forward and reverse primer (10 pmol), 22 μL of ddH_2_O, and 2 μL of DNA. The thermal cycling conditions were as follows: initial denaturation at 95°C for 7 min, followed by 35 cycles of denaturation at 95°C for 25 s and annealing at 60°C for 25 s, with a final extension step at 72°C for 30 s. Sanger sequencing of the PCR products was performed on an automated ABI PRISM 3130XL system (Applied Biosystems). The identified mutation was also examined in the parents to assess carrier status and perform segregation analysis. Finally, the sequencing results were aligned with the reference sequence obtained from the NCBI database using Chromas software.

## 3 Results

### 3.1 Clinical Description

A male neonate was born at 36 weeks of gestation (WG) due to premature labor via spontaneous vaginal delivery, presenting with harlequin ichthyosis. The newborn had a birth weight of 2800 g and Apgar scores of 4 at 1 min and 6 at 5 min. No family history of relevant medical conditions was reported, and the parents were in good health without hyperkeratotic skin lesions. Initial laboratory investigations revealed a decreased white blood cell count (5.6 × 10^9^/L; normal range: 7.7–14 × 10^9^/L) and elevated CRP levels. The patient presented with characteristic cutaneous manifestations of harlequin ichthyosis, including thick, diamond‐shaped plates covering the entire body, separated by deep fissures, that were particularly pronounced over joint areas. These severe skin findings were accompanied by several associated abnormalities: marked ectropion and eclabium, multiple joint contractures, hypoplastic fingers, and complete absence of nails (anonychia). Supportive care included skin moisturization, broad‐spectrum antibiotics for infection prophylaxis, and respiratory support. Oral retinoid (acitretin) was not initiated due to rapid clinical deterioration, which led to early neonatal death. Unfortunately, after 7 days at the Amir‐Kabir Hospital, the infant experienced respiratory distress and sepsis, ultimately resulting in death.

### 3.2 Variant Analysis

**FIGURE 2:**
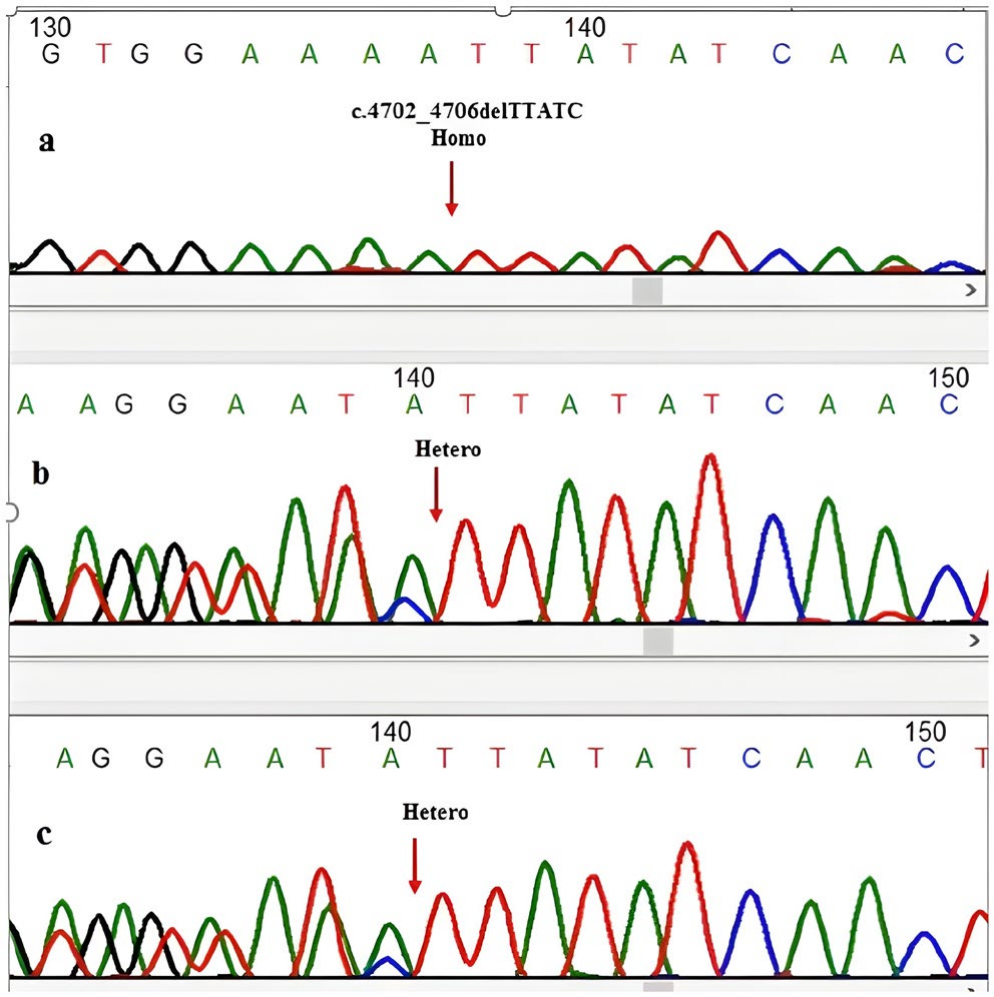
Sanger sequencing chromatograms of the *ABCA12* c.4702_4706del region. (a) Proband (homozygous mutant). (b) Father (heterozygous carrier). (c) Mother (heterozygous carrier). The deleted sequence (5 bp) is highlighted with arrow.

Whole Exome Sequencing (WES) revealed a novel homozygous mutation, NM_015657.4(ABCA12):c.4702_4706del, p.(Leu1568IlefsTer5) in the *ABCA12* gene. The mutation c.4702_4706del was not listed in the HGMD, 1000 Genomes, gnomAD, NCBI, NHLBI, and ExAC databases, highlighting the novelty and significance of this finding in genetic research. Sanger sequencing confirmed the WES findings (Figure 2a). Both parents were heterozygous carriers of the mutation (Figure 2b,c). The parents did not exhibit any signs or symptoms associated with the disease. According to the HGMD database (accessed October 15, 2024), 324 *ABCA12* variants have been linked to harlequin ichthyosis; however, c.4702_4706del has not been reported previously (https://www.hgmd.cf.ac.uk/ac/gene.php?gene=ABCA12&accession=CM001970). The variant was classified as “Likely Pathogenic” according to the ACMG guidelines, based on the following criteria: PVS1, as the mutation is a frameshift deletion expected to cause loss of function via a truncated protein; PM2, due to its absence in population frequency databases including gnomAD and the 1000 Genomes Project; PP1, based on its co‐segregation with the disease phenotype in an autosomal recessive pattern within the family; and PP4, given that the patient's clinical presentation is highly specific and consistent with the classic harlequin ichthyosis phenotype.

## 4 Discussion

Harlequin ichthyosis (OMIM 242500) is the most severe manifestation of a geno‐dermatological disorder, characterized by defects in the formation of intercellular lipid layers. This malfunction compromises barrier function, leading to clinical complications such as hyperkeratosis [19]. Historically, most neonates died within 48 h due to sepsis, respiratory failure, and electrolyte imbalances [23].

Feature abnormalities include severe ectropion, eclabium, microcephaly, joint deformities or contractures, small and swollen hands and feet, syndactyly, hypoplastic fingers, polydactyly, anonychia, underdeveloped external parts of the nose and ears, fusion of the ears to the head, open mouth, restricted joint mobility, and limited flexibility. Patients with HI experience difficulties in essential activities, including eating, breathing, and maintaining hydration, as well as increased susceptibility to infections and electrolyte imbalances, including dyselectrolemia [24, 25]. Our case underscores that severe, early‐onset sepsis and respiratory complications can still lead to fatal outcomes, particularly in resource‐limited settings or without immediate, aggressive intervention. This highlights the critical need for rapid diagnosis and multidisciplinary management. Moreover, our patient's phenotype—including thick plates, deep fissures, severe ectropion and eclabium, and joint contractures—aligns with classic severe HI presentations. However, the absence of certain features, such as polydactyly or microcephaly, in our case illustrates the phenotypic variability observed even among severe cases.

The ABCA12 gene has been identified as the causative factor of harlequin ichthyosis (HI) and can also result in congenital ichthyosiform erythroderma (CIE) or lamellar ichthyosis (LI), depending on the specific mutation. The most common mutations associated with HI are nonsense and frameshift mutations in the highly conserved regions of the ABCA12 protein, which produce truncated, nonfunctional proteins. Conversely, missense mutations in ABCA12 typically result in milder clinical manifestations, including CIE or LI [7, 16]. The identified mutation (c.4702_4706del) is a frameshift deletion predicted to produce a truncated, nonfunctional protein, consistent with the severe HI phenotype.

In 2010, a study was performed in 66 unrelated families of African, European, Pakistani/Indian, and Japanese origin, including 8 CIE, 10 LI, and 48 HI families, identifying 56 *ABCA12* mutations (online database: http://www.derm‐hokudai.jp/ABCA12): 36% nonsense, 25% missense, 4% insertion, 11% splice‐site, 5% large deletions, and 19% small deletions. No apparent mutation hotspot exists in the *ABCA12* gene; however, mutations associated with the LI phenotype are clustered within the first ATP‐binding cassette region [7, 11]. Our discovery expands this mutation spectrum and highlights that, despite the absence of a clear hotspot, frameshift deletions in critical regions remain a common pathogenic mechanism for HI.

In a study by Hotz et al. on 64 patients, 49 patients presented with identifiable phenotypes—21 HI, 20 CIE, and 8 LI—while 15 patients could not be classified according to the three main phenotypes. Mutation analysis of the *ABCA12* gene in these 64 patients identified 62 mutations: 22 (35.5%) were missense, 17 small deletions or duplications (27.5%) were predicted to induce a frameshift and premature stop codon, 14 (22.5%) were nonsense mutations resulting in a stop at a specific amino acid position, and 9 (14.5%) were splice‐site mutations predicted to affect splicing [26]. Several mutations were notably more prevalent in this study, including c.4139A>G, p.(Asn1380Ser), located in the Walker A motif of the first ATP‐binding cassette with 25 alleles, and c.4541G>A, p.(Arg1514His), with 11 alleles [26]. Notably, most patients carrying the c.4139A>G, p.(Asn1380Ser) mutation were from the Morocco/Algeria region [26].

To better understand this, we performed a comprehensive review of the literature on *ABCA12* mutations in patients with HI and their associated phenotypes. In our review, we identified a total of 70 patients carrying *ABCA12* gene variants. Among these individuals, 27 were homozygous carriers. A subset of these carriers harbored pathogenic variants affecting the consensus splice sites and adjacent regions, leading to their classification as HI patients (patients 11–16) [7, 18, 24, 27, 28]. Interestingly, only two HI patients were compound heterozygous for different splice‐site variants (patients 10 and 40) [29, 30]. Furthermore, 10 of 72 patients were classified as compound heterozygous, carrying one *ABCA12* splice‐site variant affecting the consensus splice site and a second truncating variant: eight nonsense variants (patients 1–7, 17–21, and 38) [17, 28, 31, 32, 33, 34, 35] and two frameshift mutations (patients 9 and 39) [4, 18, 36, 37]. Notably, the majority of patients had at least one truncating variant in one of their two alleles (patients 30–72) [4, 18, 26, 27, 28, 30, 35, 36, 37, 38, 39, 40, 41, 42, 43, 44, 45, 46, 47, 48]. Additionally, two HI patients carried missense variants in both alleles (patients 25 and 73) [4, 36]. Given the autosomal recessive inheritance pattern, genetic counseling is essential for carrier parents. Identifying the causative mutation enables prenatal diagnosis (via chorionic villus sampling or amniocentesis) or preimplantation genetic testing (PGT) in future pregnancies, providing families with informed reproductive options.

## 5 Conclusion

We report a case of HI in an Iranian infant, in whom a novel pathogenic ABCA12 mutation, c.4702_4706del (p.Leu1568IlefsTer5), was identified through whole‐exome sequencing. Next‐generation sequencing (NGS) is a powerful tool for diagnosing autosomal recessive congenital ichthyosis (ARCI). Identifying heterozygous carriers in consanguineous families is crucial for facilitating preimplantation genetic testing (PGT) and informed family planning. These findings clarify the molecular etiology in this patient and expand the known mutational spectrum of ABCA12.

## Author Contributions

**Nadia Soltani:** data curation, formal analysis, methodology, resources, supervision, validation, writing – original draft, writing – review and editing. **Zahra Bayati:** conceptualization, methodology, resources, writing – original draft, writing – review and editing. **Mohsen Soosanabadi:** data curation, formal analysis, investigation, resources, software, validation, writing – original draft. **Akbar Zamani:** data curation, investigation, resources, validation, visualization, writing – review and editing. **Asghar Lotfi:** data curation, investigation, methodology, resources, validation, writing – review and editing. **Milad Gholami:** conceptualization, data curation, investigation, methodology, project administration, resources, software, validation, visualization, writing – review and editing.

## Funding

The Arak University of Medical Sciences.

## Conflicts of Interest

The authors declare no conflicts of interest.

## Data Availability

The data supporting this study's findings are accessible to the corresponding author upon reasonable request.
